# Safety culture in the operating room: translation, validation of the safety attitudes questionnaire – operating room version

**DOI:** 10.1186/s12913-023-09539-9

**Published:** 2023-05-16

**Authors:** Teodor Pevec, Tit Albreht, Eva Turk

**Affiliations:** 1 General Hospital Dr. Jožeta Potrča Ptuj, Ptuj, Slovenia; 2 Medical Faculty, University of Maribor, Maribor, Slovenia; 3 National Institute of Public Health, Ljubljana, Slovenia; 4 Centre for Digital Health and Social Innovation, University of Applied Sciences St. Pölten, St. Pölten, Austria

**Keywords:** Safety culture, Operating room, Safety attitudes questionnare, Translation

## Abstract

**Background:**

Measuring the safety culture in Healthcare is an important step in improving patient safety. One of the most commonly used instruments to measure the safety climate is the Safety Attitudes Questionnaire (SAQ). The aim of the current study was to establish the validity and reliability of the Slovenian version of the SAQ for the operating room SAQ-OR.

**Methods:**

The SAQ, consisting of six dimensions, was translated and adapted to the Slovenian context and applied in operating rooms from seven out of ten Slovenian regional hospitals. Cronbach’s alpha and confirmatory factor analysis (CFA) was used to evaluate the reliability and validity of the instrument.

**Results:**

The sample consisted of 243 health care professionals who hold positions in the OR, divided into 4 distinct professional classes, namely, 76 surgeons (31%), 15 anesthesiologists (6%), 140 nurses (58%) and 12 auxiliary persons (5%). It was observed a very good Cronbach's alpha (0.77 to 0.88). The CFA and its goodness-of-fit indices (CFI 0.912, TLI 0.900, RMSE 0.056, SRMR 0.056) showed an acceptable model fit. There are 28 items in the final model.

**Conclusions:**

The Slovenian version of the SAQ-OR revealed good psychometric properties for studying the organisational safety culture.

## Background

Patient safety is a crucial aspect of healthcare provision [1]. A strong patient safety culture is important for patient safety and creating such a culture is one of the most important strategies for determining and improving patient safety in health institutions [2, 3]. A positive patient safety culture is an essential aspect of reducing errors and improving patient outcomes [4]. There are many definitions of safety culture, both within and outside healthcare [5]. Safety culture is the product of individual and group values, attitudes, competencies and behaviours that form a strong foundation on which to build a learning organization [6].

Safety culture refers mostly to individual and group values, attitudes, perceptions, and competencies with regard to safety. On the other hand, safety climate is mainly used to describe the expressed ideas, tools and techniques used in general by the organization in order to confirm its compliance with safety. In other words, the climate can easily be perceived by others, while culture is the basis that lies hidden beneath the surface. An operating room (OR) can have a high prevalence of errors, being an interdisciplinary, complex activity with a strong dependence on technical skill, where ergonomics and organizational factors play an essential role [7]. Measuring the safety climate in such a workplace is an important step in understanding patient safety [8].

An instrument that measures healthcare professionals' attitudes towards the safety climate in the OR would be helpful in understanding and identifying areas that need improvement, and for evaluating improvements in interventions [8]. The attitudes of health professionals are instantaneous snapshots of the safety cultures, reflecting the weak points and potential hazards in the medical system.

The purpose of the present study was to establish the reliability and validity of the in Slovenian translated version of the SAQ (OR version).

## Methods

### The Safety Attitudes Questionnaire (SAQ)

The SAQ was developed to measure attitudes towards the safety climate of a workplace. The SAQ is a refinement of the Intensive Care Unit Management Attitudes Questionnaire (ICUMAQ) [9], which was derived from the Flight Management Attitudes Questionnaire (FMAQ) [10]. The FMAQ was created after most airline accidents were found to be due to breakdowns in the interpersonal aspects of the crew’s performance. The SAQ was adopted for use in intensive care units. The SAQ-OR contains 30 items belonging to six dimensions [8]: teamwork climate, safety climate, job satisfaction, stress recognition, perceptions of management, and working conditions. Each item is answered using a five-point Likert scale, from “Disagree Strongly” to “Agree Strongly”, based on the respondent’s experiences in the OR department where they work.

### Translation of the Safety Attitudes Questionnaire – Operating Room (SAQ-OR)

The questionnaire was translated from the English original in Göras et al.’s article [8]. The EN-SLO translation was made by a Slovene native speaker with a high degree of fluency in English.

After this, another expert in English translated the questionnaire back from Slovene into English. Finally, we compared the original version and our back translated version. We have modified just a few words.

### Face validity

The face validity was tested by three physicians and one nurse, who were selected randomly from the OR team and were from different age groups and specialities. The questionnaire was thus reviewed by a surgeon and an anaesthesiologist with more than 10 years experience, a resident with three years experience, and an OR nurse with more than 10 years experience. They were asked to give feedback about the comprehensibility and minimal changes to the questionnaire were made to their oral feedback.

### Data collection and ethical considerations

A cross-sectional design was used to test the questionnaire. Surgeons, nurses, anaesthesiologists and auxiliary nurses from seven of the ten Slovenian regional hospitals, all with at least 1 year of OR work experience, were asked to fill in the Slovene translation of the SAQ-OR (written questionnaires). We chose hospitals where we had such good personal contacts with the managers that we could ensure the appropriateness of conducting the research at the hospital level. At the team meetings, the purpose of the questionnaire was explained, and potential participants were asked to fill out the questionnaire. We guaranteed that participation was voluntary and anonymous. We sent the questionnaires to the medical directors by post or delivered them in person.

The respondents were informed that participation was voluntary. The questionnaires were treated anonymously. The response rate of completed questionnaires was 65%, so there were 243 participants (from 374 original requests).

The present study respected the bioethical principles for medical research on human beings of the Declaration of Helsinki, related to confdentiality, freedom, respect and non-malefcence; and, was approved by the Hospital dr. Jožeta Potrča, Ptuj Ethics Committee (01/3–81/11–22).

### Statistical analysis

Initially, descriptive statistics used frequencies (*n*) and percentages (%) to summarize nominal and ordinal data. The internal consistency of the total SAQ-OR and its six factors, *teamwork climate*, *safety climate, job satisfaction, stress recognition, perception of management, and working conditions*, was measured by calculating Cronbach's alpha. Confirmatory Factor Analysis (CFA) was applied to test the validity of the SAQ. The CFA is also sufficient to verify the theoretically-founded factors as well as to identify factor loadings and fit of single items per factor/to identify certain items that should be excluded.

IBM SPSS Statistics 20, IBM Amos, JASP and the Mplus software package Version 7.32 were used, using maximum likelihood estimation with robust standard errors (MLR), which is robust to the deviation of scores from the normal distribution and the dependence of observations. Several fit indices were used to evaluate the model: the Yuan-Bentler χ^2^ test (YBχ^2^), the Root Mean Square Error of Approximation (RMSEA), the Comparative Fit Index (CFI), the Taker-Lewis Index (TLI), and the Standardized Root Mean Squared Residual (SRMR). The Yuan-Bentler χ^2^ should be statistically insignificant to indicate that the model fits the data well; however, with large samples, a non-significant value of the χ^2^ test is very rarely obtained [11]. A value of the RMSEA less than 0.06, a value of the SRMR below 0.08, and values of the Comparative Fit Index and the Tucker-Lewis Index greater than 0.95 indicate a good model fit [11]. The criteria for an acceptable model fit are as follows: RMSEA between 0.06 and 0.08, SRMR between 0.08 and 0.10, and CFI and TLI above 0.90 [12].

## Results

No significant differences were detected between the translations, and no major remarks were made during face validity.

The sample consisted of 243 healthcare professionals who hold positions in the OR, divided into 4 distinct professional classes: 76 surgeons (31%), 15 anaesthesiologists (6%), 140 nurses (58%) and 12 auxiliary personnel (5%).

The participants had a mean age of 41.5 years, with a minimum of 25 years and maximum of 65 years. The average number of years that the health professionals had worked in that institution was 13.1. With regard to the sex distribution of the sample, 79 were male (33%) and 164 were female (67%). The SAQ dimensions, items, factor loadings and Cronbach`s alpha for items are presented in Table 1. The dimension *Job satisfaction* received the highest average grade (4.1) and the dimension *Perceptions of Management* the lowest average grade (3.1). Two questions (in bold in Table 1) (“In this clinical area, it is difficult to speak up if I perceive a problem with patient care”, and “In this clinical area, it is difficult to discuss errors”) had insufficient factor loadings and we had to exclude them.

**Table 1:** Slovenian version of the SAQ- OR and item descriptives

**Teamwork Climate /Vzdušje v operacijski dvorani**	**Mean (SD)** **3.8 (0.7)**	**Loadings**	**Cronbach's Alpha if item deleted**
1. Nurse input is well received in this clinical area. *Delo medicinskih sester v operacijskih dvoranah je tukaj dobro sprejeto*	(0.9)	0.52	0.60
2. **In this clinical area, it is difficult to speak up if I percieve a problem with patient care** **V operacijskih dvoranah je težko spregovoriti o zaznanih težavah pri oskrbi bolnikov**	2.7 (1)	-0.19	0.82
3. Disagreements in this clinical area are resolved appropriately *V operacijskih dvoranah se nesoglasja ustrezno rešujejo (ne kdo ima prav, ampak kaj je najbolje za bolnika)*	3.3 (1)	0.79	0.56
4. I have the support I need from other personnel to care for patients *Drugi sodelavci mi dajejo podporo, ki jo potrebujem za oskrbo bolnikov v operacijskih dvoranah*	4 (0.9)	0.55	0.59
5. It is easy for personnel here to ask questions when there is something that they do not understand *Zaposleni v operacijskih dvoranah lahko brez težav vprašajo, kadar česa ne razumejo*	4 (0.9)	0.63	0.57
6. The physicians and nurses here work together as a well-coordinated team *Zdravniki in sestre tukaj delajo skupaj kot dobro usklajena ekipa*	3.8 (0.9)	0.65	0.52
**Safety Climate /Varnostno vzdušje v operacijski dvorani**	**3.8 (0.7)**	**Loadings**	**Cronbach`s Alpha if item deleted**
7. I would feel safe being treated here as a patient *Če bi se tukaj zdravil kot bolnik, bi se počutil varno*	4 (0.9)	0.64	0.66
8. Medical errors are handled appropriately in this clinical area *Zdravstvene napake v operacijskih dvoranah se ustrezno obravnavajo*	3.9 (0.9)	0.71	0.64
9. I know the proper channels to direct questions to regarding patient safety in this clinical area *Poznam primerne poti za naslavljanje vprašanj glede varnosti bolnikov v operacijskih dvoranah*	3.8 (0.9)	0.71	0.64
10. I receive appropriate feedback about my performance *O uspešnosti svojega dela prejemam ustrezne povratne informacije*	3.6 (1.1)	0.71	0.65
11. **In this clinical area, it is diffcult to discuss errors** ***V operacijskih dvoranah se je težko pogovoriti o napakah***	2.7 (1.1)	-0.15	0.84
12. I am encouraged by my colleagues to report any patient safety concerns I may have *Sodelavci me spodbujajo, da poročam o svojih morebitnih skrbeh glede varnosti bolnikov*	3.6 (0.9)	0.54	0.65
13. The culture in this clinical area makes it easy to learn from the errors of others *Kultura v operacijskih dvoranah mi omogoča, da se lahko učim iz napak drugih*	3.7 (0.9)	0.60	0.65
**Job Satisfaction /Zadovoljstvo z delom v operacijski dvorani**	**4.1 (0.7)**	**Loadings**	**Cronbach`s Alpha if item deleted**
14. I like my job *Rad/-a imam svoje delo*	4.5 (0.7)	0.54	0.81
15. Working here is like being part of a large family *Ko delam v operacijski dvorani, se počutim kot del velike družine*	3.9 (0.9)	0.68	0.81
16. This is a good place to work *Delo v operacijski dvorani je dober poklic*	4.3 (0.8)	0.63	0.78
17. I am proud to work in this clinical area *Ponosen/-a sem, da delam v operacijski dvorani*	4.3 (0.8)	0.68	0.76
18. Morale in this clinical area is high *Morala v operacijskih dvoranah, kot delovnem okolju, je visoka*	3.7 (0.9)	0.57	0.81
**Stress Recognition /Prepoznavanje stresa pri delu v operacijski dvorani**	**3.6 (1)**	**Loadings**	**Cronbach`s Alpha if item deleted**
19. When my workload becomes excessive, my performance is impaired *Pri preveliki delovni obremenitvi je moja delovna uspešnost slabša*	3.6 (1.1)	0.88	0.85
20. I am less effective at work when fatigued *Kadar sem preutrujen/-a, sem manj učinkovit/-a pri svojem delu*	3.9 (1)	0.91	0.83
21. I am more likely to make errors in tense or hostile situations *Verjetneje je, da bom v napetih in sovražnih situacijah delal/-a napake*	3.8 (1.1)	0.90	0.83
22. Fatigue impairs my performance during emergency situations (e.g. emergency resuscitation, seizure) *Utrujenost poslabša mojo delovno uspešnost v nujnih primerih (npr. krvavitev med operacijo)*	3.4 (1.3)	0.99	0.84
**Perceptions of Management /Razumevanje dela v operacijski dvorani s strani vodstva**	**3.1 (0.8)**	**Loadings**	**Cronbach`s Alpha if item deleted**
23. Hospital management supports my daily efforts *Vodstvo bolnišnice podpira moje vsakodnevne napore*	2.9 (1.1)	0.89	0.67
24. Hospital management does not knowingly compromise the safety of patients *Vodstvo bolnišnice zavestno ne ogroža varnosti bolnikov v operacijskih dvoranah*	3.6 (1.0)	0.65	0.68
25. The levels of staffing in this clincal area are sufficient to handle the number of patients. *Kadrovska struktura v operacijski dvorani je zadostna za obravnavo dotičnega števila bolnikov*	2.8 (1.0)	0.61	0.78
26. I get adequate, timely information about events that might affect my work from hospital management *Od vodstva dobim ustrezne in pravočasne informacije o dogodkih v operacijskih dvoranah, ki bi lahko vplivali na moje delo*	3.2 (1.0)	0.84	0.70
**Working Conditions /Pogoji dela v operacijskih dvoranah**	**3.5 (0.7)**	**Loadings**	**Cronbach`s Alpha if item deleted**
27. This hospital does a good job of training new personnel *V operacijskih dvoranah se nov kader dobro usposobi*	3.5 (0.9)	0.72	0.7
28. All necessary information for diagnostic and therapeutic decisions is available to me routinely *Vse potrebne informacije za odločitve glede strokovnega dela so mi v operacijskih dvoranah vedno na voljo*	3.7 (0.9)	0.68	0.80
29. Problem personnel are dealt with constructively by our hospital management *V operacijskih dvoranah konstruktivno obravnavamo težavno osebje*	3.2 (1.0)	0.58	0.79
30. Trainees in my discipline are supervised adequately *Učeče se osebje je v operacijskih dvoranah pod primernim nadzorom*	3.6 (0.9)	0.66	0.71

The questions that was excluded are marked in bold

In order to study the internal consistency of the instrument used, Cronbach's alpha for each of the factors of the questionnaire was calculated. We obtained a very good Cronbach`s alpha (0.77 to 0.88).

The original six-factor solution (YBχ^2^(335) = 639,23, *p* < 0.001; CFI = 0.895, TLI = 0.882, SRMR = 0.064; RMSEA = 0.061, 90% CI = 0.054, 0.064) showed good or acceptable model fit in the case of RMSEA and SRMR fit indices, however, the values of CFI and TLI indices were slightly lower than the acceptable values. The six-factor model was modified by correlating errors for items with similar/same meaning, which was suggested by high values of modification indices (over 30) (items 1 and 4 in the job satisfaction subscale and items 1 and 4 in the working condition subscale). Fit indices of the modified six-factor model (YBχ^2^(333) 588.93, *p* < 0.001; CFI = 0.912, TLI = 0.900, SRMR = 0.056; RMSEA = 0.056, 90% CI = 0.049, 0.064) indicate good fit for RMSEA and SRMR, as well as acceptable fit for TLI and CFI. Item factor loadings and factors' Cronbach alpha of the final modified six—factor are presented in Table 2.

**Table 2:** Item factor loadings and factors' Cronbach alpha of the final modified six-factor

Factor	Items	Factor loading	Cronbach`s alpha
Teamwork Climate Vzdušje v operacijski dvorani			0.82
	1. Nurse input is well received in this clinical area *Delo medicinskih sester v operacijskih dvoranah je tukaj dobro sprejeto*	0.52	
	2. Disagreements in this clinical area are resolved appropriately *V operacijskih dvoranah se nesoglasja ustrezno rešujejo (ne kdo ima prav, ampak kaj je najbolje za bolnika)*	0.78	
	3. I have the support I need from other personnel to care for patients *Drugi sodelavci mi dajejo podporo, ki jo potrebujem za oskrbo bolnikov v operacijskih dvoranah*	0.56	
	4. It is easy for personnel here to ask questions when there is something that they do not understand *Zaposleni v operacijskih dvoranah lahko brez težav vprašajo, kadar česa ne razumejo*	0.63	
	5. The physicians and nurses here work together as a well-coordinated team *Zdravniki in sestre tukaj delajo skupaj kot dobro usklajena ekipa*	0.65	
Safety Climate Varnostno vzdušje v operacijski dvorani			0.84
	6. I would feel safe being treated here as a patient *Če bi se tukaj zdravil kot bolnik, bi se počutil varno*	0.64	
	7. Medical errors are handled appropriately in this clinical area *Zdravstvene napake v operacijskih dvoranah se ustrezno obravnavajo*	0.71	
	8. I know the proper channels to direct questions to regarding patient safety in this clinical area *Poznam primerne poti za naslavljanje vprašanj glede varnosti bolnikov v operacijskih dvoranah*	0.71	
	9. I receive appropriate feedback about my performance *O uspešnosti svojega dela prejemam ustrezne povratne informacije*	0.71	
	10. I am encouraged by my colleagues to report any patient safety concerns I may have *Sodelavci me spodbujajo, da poročam o svojih morebitnih skrbeh glede varnosti bolnikov*	0.54	
	11. The culture in this clinical area makes it easy to learn from the errors of others *Kultura v operacijskih dvoranah mi omogoča, da se lahko učim iz napak drugih*	0.60	
Job Satisfaction Zadovoljstvo z delom v operacijski dvorani			0.83
	12. I like my job *Rad/-a imam svoje delo*	0.54	
	13. Working here is like being part of a large family *Ko delam v operacijski dvorani, se počutim kot del velike družine*	0.68	
	14. This is a good place to work *Delo v operacijski dvorani je dober poklic*	0.63	
	15. I am proud to work in this clinical area *Ponosen/-a sem, da delam v operacijski dvorani*	0.68	
	16. Morale in this clinical area is high *Morala v operacijskih dvoranah, kot delovnem okolju, je visoka*	0.57	
Stress Recognition Prepoznavanje stresa pri delu v operacijski dvorani			0.88
	17. When my workload becomes excessive, my performance is impaired *Pri preveliki delovni obremenitvi je moja delovna uspešnost slabša*	0.88	
	18. I am less effective at work when fatigued *Kadar sem preutrujen/-a, sem manj učinkovit/-a pri svojem delu*	0.91	
	19. I am more likely to make errors in tense or hostile situations *Verjetneje je, da bom v napetih in sovražnih situacijah delal/-a napake*	0.90	
	20. Fatigue impairs my performance during emergency situations (e.g. emergency resuscitation, seizure) *Utrujenost poslabša mojo delovno uspešnost v nujnih primerih (npr. krvavitev med operacijo)*	0.99	
Perceptions of Management Razumevanje dela v operacijski dvorani s strani vodstva			0.77
	21. Hospital management supports my daily efforts *Vodstvo bolnišnice podpira moje vsakodnevne napore*	0.90	
	22. Hospital management does not knowingly compromise the safety of patients *Vodstvo bolnišnice zavestno ne ogroža varnosti bolnikov v operacijskih dvoranah*	0.65	
	23. The levels of staffing in this clincal area are sufficient to handle the number of patients *Kadrovska struktura v operacijski dvorani je zadostna za obravnavo dotičnega števila bolnikov*	0.61	
	24. I get adequate, timely information about events that might affect my work from hospital management *Od vodstva dobim ustrezne in pravočasne informacije o dogodkih v operacijskih dvoranah, ki bi lahko vplivali na moje delo*	0.84	
Working Conditions Pogoji dela v operacijskih dvoranah			0.80
	25. This hospital does a good job of training new personnel *V operacijskih dvoranah se nov kader dobro usposobi*	0.72	
	26. All necessary information for diagnostic and therapeutic decisions is available to me routinely *Vse potrebne informacije za odločitve glede strokovnega dela so mi v operacijskih dvoranah vedno na voljo*	0.68	
	27. Problem personnel are dealt with constructively by our hospital management *V operacijskih dvoranah konstruktivno obravnavamo težavno osebje*	0.58	
	28. Trainees in my discipline are supervised adequately *Učeče se osebje je v operacijskih dvoranah pod primernim nadzorom*	0.66	

Fit indices of the modified six – factor model: YBχ2(333) 588.93, *p* < 0.001; CFI = 0.912, TLI = 0.900, SRMR = 0.056; RMSEA = 0.056, 90% CI = 0.049, 0.064

The intercorrelations between the factors are presented in Table 3. All subscales have moderate to high positive correlations with each other, except for the stress recognition subscale, with which the other subscales achieves low negative or nonsignificant correlations.

**Table 3:** Correlations between factors: the modified six-factor solution (*n* = 243)

	1	2	3	4	5	6
1. Teamwork climate subscale	1					
2. Safety climate subscale	.867^**^	1				
3. Job satisfaction subscale	.598^**^	.671^**^	1			
4. Stress recognition subscale	-.180	-.283^*^	-.246^*^	1		
5. Perc. of management subscale	.566^**^	.670^**^	.488^**^	-.188	1	
6. Working condition subscale	.687^**^	.679^**^	.532^**^	-.149	.646^**^	1

^**^p < .01; ^*^p < .05

## Discussion

This study evaluated the psychometric properties of the 28-items SAQ-OR Slovenian-language version. The results showed good Cronbach’s alpha and acceptable CFA fit statistics. The present study represents the first report on attitudes relevant to safety culture in ORs in Slovenia. Klemenc-Ketis et al. [13] presented a study in which they developed the SAQ for our country, but it was for the out-of-hours primary care setting.

The development of a valid and reliable instrument is a longitudinal and multi-step process [13]. We chose one of the most widely used surveys (the SAQ-OR) [14] and included all the professional profiles involved in the work of the OR (anaesthesiologists, surgeons, operating room nurses, auxiliary personnel) in most Slovenian regional hospitals. Work in the OR must be conducted as a team, and all personnel must be aware of the importance of a safety culture.

The number of participants in the study was sufficient (*n* = 243) for the model fit to be good. There was also a good proportion of returned questionnaires (65%), and it was implemented in seven of the ten regional hospitals in Slovenia. Göras et al. [8] tested the Swedish SAQ in three hospitals with 237 respondents (and 63% of the questionnaires were returned). Bernalte-Marti [15] in Spain and in Italy made validated questionnaire with only 30 experts, while Pinheiro et Uva [7] carried out their study at one hospital in Portugal with 82 healthcare professionals.

The questionnaire consisted of six dimensions [16]. We hoped to include as many areas of risk for patient safety as possible. The dimension *Job satisfaction* received the highest average rating, and the dimension *Perceptions of Management* received the lowest average rating. The average response values were very high for claims of *Job satisfaction* working in the OR (for example, 4.4 for *“This is a good place to work”*). Pinheiro and Uva [7] also found the highest average rating to be in the *Job satisfaction* dimension and the lowest average rating in the dimension *Perception of Management*. Job satisfaction is related to fewer adverse events [17].

Low management perceptions suggest that professionals do not notice any commitment from the management with regard to safety culture (item *Hospital management supports my daily efforts –*rating 2.9). Our country also constantly deals with a lack of suitable personnel (item *The levels of staffing in this clinical area are sufficient to handle the number of patients*—rating 2.8). Carvalho and al. [18] in their Brazilian study also noted a poor result in the dimension Hospital management.

We showed that the questionnaire has appropriate internal consistency (Cronbach’s alpha values between 0.77 and 0.88). In a Swedish study of psychometric testing of the same questionnaire, Göras et al. [8] demonstrated a Cronbach’s alpha between 0.59 and 0.83, while in Portugal, Pinheiro and Uva [7] reported a Cronbach’s alpha between 0.34 and 0.72, and in Italy and Spain, a study carried out by Bernalte-Marti [15] reported a Cronbach’s alpha between 0.37 and 0.78. The CFA performed on the 6-dimensional model confirmed that the model is good and two questions did not meet all the strict criteria of the factor analysis and were therefore excluded from the final questionnaire. Methodologically, the approaches of other authors were similar. Pinheiro and Uva [7] and Göras et al. [8] measured Cronbach's alpha and passed the CFA but Bernalte-Marti [15] Marti first performed a Cronbach’s alpha, and in the next study [19] carried out an EFA and CFA on the same sample.

The exclusion of two questions is a special feature of our research. Obviously, the factors that determine the safety culture in our country are different than elsewhere, but the purpose of the validation is to adjust the questionnaire to the cultural and other characteristics of our country.

This is in concordance with the Harzing [20] finding, that questionnaires in the english language elicit a higher rate of an intermediate responses, while the questionnaires in the respondent's native language elicit more extreme response styles.

### Limitations of the study

A pool of different professionals was included in our study, and given the subsample sizes, further sub-analysis to test for possible differences in responses and factor structure between the professions was hardly feasible. Also the sampling procedure, in so many different hospitals, could have had some bias. The study was limited to self-reported outcome. There was no continuous assessment of safety attitudes over time.

## Conclusions

The Slovenian version of the SAQ-OR demonstrates good psychometric capabilities for studying the safety culture of a workplace. The questionnaire seems to have potential as a useful tool for evaluating safety culture. Health professionals are satisfied with their work, but management has not promoted patient safety.

## Data Availability

The datasets used and/or analyzed during the current study are available from the corresponding author on reasonable request.
